# Knowledge, attitude and practice towards COVID-19 among secondary school students in Gondar town, Northwest Ethiopia

**DOI:** 10.1371/journal.pone.0268084

**Published:** 2022-05-23

**Authors:** Solomon Getawa, Melak Aynalem, Biruk Bayleyegn, Tiruneh Adane

**Affiliations:** Department of Hematology and Immunohematology, School of Biomedical and Laboratory Sciences, College of Medicine and Health Science, University of Gondar, Gondar, Ethiopia; Management and Science University, MALAYSIA

## Abstract

**Background:**

In Ethiopia, an array of measures have been adopted to control the rapid spread of the Coronavirus Disease 2019 (COVID-19) pandemic. Such control measures could significantly influence the knowledge, attitudes, and practices (KAP) towards COVID-19 in the general population. However, still, there is scarce information regarding the KAP of students towards the COVID-19 pandemic. Therefore, this study aimed to assess KAP and associated factors towards COVID-19 among secondary school students in Gondar town, Ethiopia.

**Methods:**

A cross-sectional study was conducted from February to April 2021 on a total of 395 participants. Proportional sample allocation was used in 4 randomly selected schools. Then, students from each of the schools were recruited by using a systematic random sampling technique. Socio-demographic data and questions regarding the KAP were collected via a self-administered questionnaire. Statistical analysis was performed by using SPSS 20. Logistic regression analyses were used to identify the associated factors and p-value <0.05 was considered statistically significant.

**Results:**

The mean age of study participants was 17.7±1.5 years and slightly more than 2/3 (67.3%) ranges from 17–19 years old. In this study, 86.3% (95% CI: 83–90) of study participants had good knowledge about COVID-19. Students having urban residence (AOR, with 95% CI: 5.6 (1.76–17.6), fathers with a diploma and above educational status (AOR, with 95% CI: 3 (1.2–7.5), and uses television or radio as a source of information (AOR, with 95% CI: 3.7 (1.5–9.3) tended to have good knowledge about COVID-19. About 381 (86.3%) had good attitude towards COVID-19 infections, while 238 (60.3%) of the participants had a good practice to prevent COVID-19 infections.

**Conclusions:**

The majority of the secondary school students in Gondar town have good knowledge, attitude, and practices towards COVID-19. However, targeted interventions are still necessary, especially for students having poor knowledge and poor practice towards COVID-19. This study also found that urban residence, a father with a diploma and above educational status, and using television or radio as a source of information about COVID-19 were significantly associated with the knowledge level of the study participants.

## Introduction

On 31st December 2019, cases of pneumonia of unknown cause in Wuhan, China were reported to the World Health Organization (WHO). The pathogen identified was severe acute respiratory syndrome coronavirus-2 (SARS-CoV-2) [1]. The viruses primarily spread through respiratory droplets of saliva or discharge from the nose when an infected person coughs or sneezes [2]. The common symptoms of Coronavirus Disease 2019 (COVID-19) include fever, dry coughing, and fatigue or serious symptoms (difficulty in breathing, chest pain, difficulty in talking, and moving, and multiple systemic illnesses) [3].

The WHO declared that the novel coronavirus outbreak is a global pandemic on March 11, 2020 [4]. The strategies established worldwide to reduce the transmission are mostly behavioral (e.g. social distancing, regular washing of hands, one’s knowledge about the problem, ability to perceive the risk, and willingness to change their attitude) [5]. In Ethiopia, the outbreak was officially confirmed on the 13^th^ of March 2020. The person found positive was a 48-year-old Japanese citizen who came to Ethiopia on March 4, 2020, from Burkina Faso [6]. As a result, the government declared a state of emergency to limit the spread of the COVID-19 [7]. As of July 13, 2021, over 186 million cases and 4 million deaths have been reported globally [8]. In Africa, there were 4, 359, 925 confirmed cases and 102, 687 deaths, with a fatality rate of 2.4 percent [9]. More than 277, 318 confirmed cases and 4,349 deaths have been reported in Ethiopia [10].

Knowledge, attitude, and practice (KAP) is an important cognitive key in public health regarding health prevention and promotion. It involves a range of beliefs about the causes of the disease and exacerbating factors, identification of symptoms, and available methods of treatments and consequences [11]. It is also a suitable way to evaluate existing programs and to identify effective strategies for behavioral change in society. In many cases, the absence of knowledge, or if most of the medical-related beliefs are misconstrued, may carry a potential risk [12].

To date, no successful anti-viral treatment has been reported. However, different vaccines are now available, even if the vaccine is not fairly distributed to developing countries including Ethiopia. As a result, applying the preventive measure to control COVID-19 infection is the utmost critical intervention [13]. Ethiopia had implemented different preventive measures including school closure, staying at home, keeping social distance, appropriate hand washing in places of common use like the bank, market, and church/mosques, and declaring it a state emergency at the national level [14, 15]. But, now the government of Ethiopia declared that schools are reopened and the education activities have been started by applying COVID-19 protective protocols. Because of the high infectivity nature of the virus, the large asymptomatic people, and lack of effective antivirals medications, the management of COVID- 19 has become very difficult [16]. There is scarce information regarding the level of awareness of school students in Ethiopia. Therefore, this study aimed to identify the current status of knowledge, attitude, and practice regarding COVID-19 among secondary school students in Gondar town.

## Materials and methods

### Study design, setting and period

A cross-sectional study was conducted among secondary school students in Gondar town from February to April 2021. The town is the capital city of the central Gondar zone and it is found 186 km away from Bahir-Dar. Bahir-Dar is the capital city of the Amhara region, that far away 328 km from Addis Ababa, the capital city of Ethiopia. It has twelve (12) sub-cities [17]. The town has 15 secondary schools; 12 governmental and 3 private schools [18].

### Study population

All students who attended secondary schools in Gondar town were recruited as source population while students who attended in the selected secondary schools in Gondar town and who are avail during the study period and willing to participate in the study were considered as the study population. All students who are following their secondary school in Gondar town were included.

### Study variables

Socio-demographic characteristics such as age, gender, residence, religion, family educational status, family occupation, and family monthly income were the independent variables whereas knowledge, attitude and practice level of the students were the dependent variables.

### Sample size and sampling techniques

The sample size was determined by using the single population proportion formula by assuming p = 50%. Then, the sample size becomes 384. By considering a 10% non-response rate, the final sample size was 422 secondary school students. Initially, four schools, such as Fasiledes secondary schools, Ediget Felege, Walliya, and Fasiledes preparatory schools, were selected using simple random sampling from the total 15 schools. Then, a systematic sampling technique was employed to select the study participants from each of the selected schools. The numbers of study participants sampled from the selected schools were determined using proportionate-to-population size. There were a total of 8,647 students in 4 selected schools: 2592 in Fasiledes secondary, 2734 in Ediget Felege, 750 in Walliya, and 2571 in Fasiledes preparatory school. The interval (*K*) value was calculated for each selected school by dividing the total number of students in each school by the corresponding proportional sample size calculated for each school. The initial participant was randomly selected by the lottery method. Then other participants were selected at every *K*^th^ interval.

### Data collection procedure

Socio-demographic information were collected using a pre-tested structured self-administered questionnaire that is adopted from the Ethiopian surveillance system for COVID-19. The questionnaire includes socio-demographic characteristics and KAP towards COVID-19. During data collection, all the necessary personal protective equipment (PPE) was applied. Socio-demographic characteristics include gender, residence, age, grade/section, religion, parental educational status, parental occupational status, family size, residence, and source of information about COVID-19.

#### Knowledge, attitude, and practice

Knowledge about COVID-19 was assessed using 23 general questions, which are deemed to assess the causative agents, ways of transmission, and prevention mechanisms. The questionnaire was answered on a true/false basis. A correct answer was assigned as “1” and an incorrect/unknown answer was assigned as “0”. The scoring ranges from 23 to 0. The scores for individuals were calculated and summed up to get the knowledge of the participants about COVID-19. Besides, the student’s attitude was assessed using 14 questions. Each question contain three responses; “Disagree”, “Neutral”, and “Agree and labeled as 0, 1, and 2, respectively. For the purpose of analysis, “Disagree and Neutral” was recoded to “0” and “Agree” was recorded to “1”. Then, the scores were summarized to get the overall attitude of the students about COVID-19. Similarly, 12 preventive practices related to COVID-19 questions were asked, and the responses of each question were scored as “1” for correct and “0” for incorrect response. The practice scoring ranges from 12 (largest) to 0 (smallest). Preventive practice scores for individuals were calculated and summed up to give the total practice score.

### Data quality control

The questionnaire was prepared in English language and back-translated to the local language Amharic to ensure its consistency. The accuracy of the tool was checked by back translating to English by experts who were blind to the original instrument. Before starting the data collection, the pre-test was done on 10% of the total sample size in “Angereb” secondary school, and an amendment was made accordingly. To maintain the quality of the data, training was provided for data collectors on the aim of the study and methods of data collection. Field level supervision was done to control the data collection and data quality.

#### Reliability and validity of the research

Cronbach’s alpha reliability coefficients were computed to determine the internal consistency of all research constructs: Cronbach’s alpha of 0.7 or above indicates high reliability, between 0.5–0.7 indicates moderate reliability and less than 0.5 indicates low reliability. The Cronbach’s alpha values for the knowledge, attitude and practice constructs were 0.53, 0.78, and 0.73, respectively.

### Data processing and analysis

The data was cleaned, checked for completeness, and entered using Epi Data-V.4.6, and exported to SPSS version 23 software for analysis. The items were first coded as “1” favoring a good outcome and “0” not favoring a poor outcome. A good score is defined as if the participant correctly responds 50% and more for KAP assessing questions [19]. Reversing it, the poor score is defined as if participant correctly respond less than 50% of KAP assessing questions [20].

Then, the data were analyzed using appropriate descriptive statistics, and summarized by frequency, percentage, and mean. Both Bivariate and multivariate logistic regression analyses were performed to identify associated factors of good KAP towards COVID-19 infection. The variables in bivariate analysis with p < 0.2 were entered into multivariate logistic regression. The model fitness was also checked using the Hosmer-Lemeshow model fit-ness test. The strength of the association was demonstrated by computing the crude odds ratio (COR) and adjusted odds ratio (AOR) with a 95% confidence interval (CI). P-value <0.05 was considered statistically significant.

## Ethical considerations

The study protocol was evaluated and approved by the institutional ethical review board of the University of Gondar and considered the Helsinki Declaration. Support letters were submitted to the selected schools of the town, and letters of permission were secured from the administrative bodies and coordinators. Then, recruited participants were given a verbal explanation about the objectives of the research and provided a written information sheet. All potential participants who agreed to participate provided written consent to continue with the interviews. For adults younger than 18 years, consent was taken from the school, and an assent form was also obtained from each participant. The confidentiality of information obtained was kept, and respondents’ names were not recorded.

## Results

### Socio-demographic characteristics of the study participants

From 422 approached students, a total of 395 study participants with a response rate of 93.6% were included in this study. The mean age of study participants was 17.7±1.5 years and slightly more than 2/3 (67.3%) ranges from 17–19 years old. Almost all (95.4%) of study participants were from urban residents and nearly two-third of the participants (62.7%) were female students (Table 1).

**Table 1:** Socio-demographic characteristics of secondary school students in Gondar town, Northwest Ethiopia, 2021.

Characteristics	Category	Frequency (n)	Percentages (%)
Age/years	14–16	79	20.0
	17–19	266	67.3
	20–23	50	12.7
Gender	Male	147	37.2
	Female	248	62.8
Grade	Grade 9 and 10	170	43.0
	Grade 11 and 12	225	57.0
Resident	Urban	377	95.4
	Rural	18	4.6
Religion	Orthodox	324	82.0
	Muslim	61	15.4
	Protestants and Catholic	10	2.5
Mother’s educational status	No education	108	27.3
	Primary/secondary	218	55.2
	Diploma and above	69	17.5
Father’s educational status	No education	106	26.8
	Primary/secondary	169	42.8
	Diploma and above	120	30.4
Mother’s occupation	Health related	19	4.8
	None- health related	69	17.5
	Housewife	250	63.3
	Others	57	14.4
Father’s occupation	Health related	24	6.1
	None- health related	97	24.6
	Not-employed	196	49.6
	Others	78	19.7
Family member >60 years old?	Yes	163	41.3
	No	232	58.7
Number of family members	≤5	179	45.3
	>5	216	54.7

#### Source of information about COVID-19

**Fig 1:**
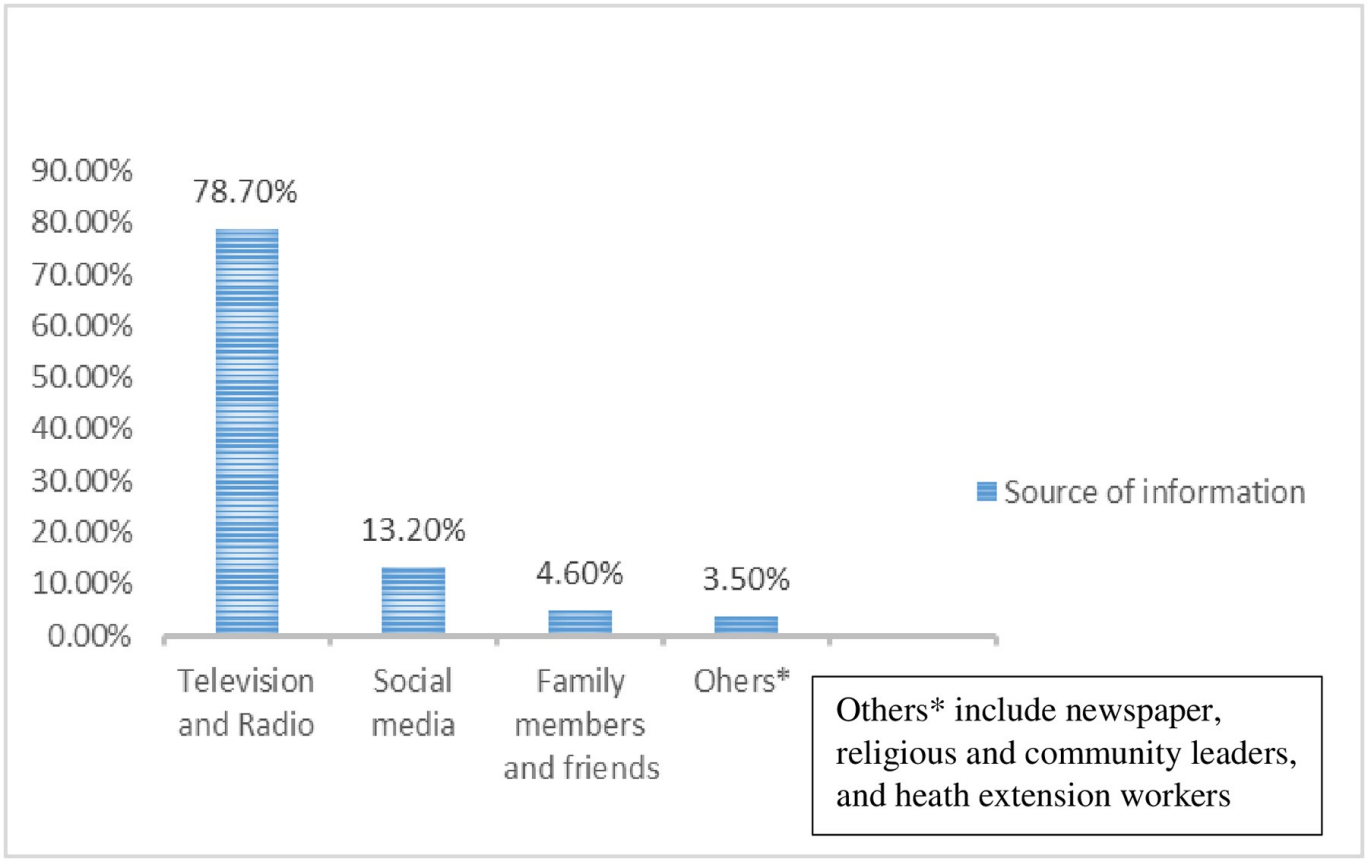
Source of information for students in central Gondar zone, Northwest Ethiopia, 2021.

As summarized in Fig 1, more than 3\4 (78.7%) of the study participants responded, mass media was the main source of information about COVID-19.

### Knowledge of students about COVID-19

In this study, about 86.3% (95% CI: 83–90%) of study participants had good knowledge about COVID-19. Almost all the study participants (94.9%) and (94.7%) were correctly answered that COVID-19 is a viral disease that affects the respiratory system and transmitted by airborne through respiratory droplets from coughing, respectively. Regarding the preventive mechanisms, 90.1% of study participants were answered correctly that wearing masks is one of the pillars of COVID-19 infection preventive methods. Moreover, 90.9% of study participants are aware that immune-compromised and chronic disease patients are at high risk of developing COVID-19 complications. Meanwhile, more than half (55.2%) of the respondents did not know that severe obesity is a high risk to develop COVID-19 complications and 49.9% had miss understanding and considered as all COVID-19 infected people may develop symptoms (Table 2).

**Table 2:** Frequency of respondents towards knowledge indicators about COVID-19 among secondary school students in Gondar town, Northwest Ethiopia, 2021.

Indicators	Yes N (%)	No N (%)
Is COVID -19 a curable disease? **	369 (93.4)	26 (6.6)
Can COVID-19 cause death? *	355 (89.9)	40 (10.1)
Is COVID-19 viral disease that affects the respiratory system? *	375 (94.9)	20 (5.1)
Is COVID-19 transmitted by touching contaminating objects or surface? *	336 (85.1)	59 (14.9)
Is COVID-19 transmitted by direct contact with patients *	362 (91.6)	33 (8.4)
Does COVID-19 transmitted by airborne including through respiratory droplets from sneezing or coughing? *	374 (94.7)	21 (5.3)
COVID-19 can be transmitted by anopheles biting? **	158 (40)	237(60)
Is COVID-19 transmitted by touching the face, eyes, nose, and mouth with contaminated hands? *	364 (92.2)	31 (7.8)
Fever, Fatigue, Cough, Body pain and Sore throat is a symptom of COVID-19 *	368 (93.2)	27 (6.8)
Diarrhea or constipation is a symptom of COVID-19 **	138 (34.9)	257 (65.1)
Are all people infected with COVID-19 will develop symptoms? **	198 (50.1)	197 (49.9)
Individuals with COVID-19 can be identified by observation? **	107 (27.1)	288 (72.9)
Is the disease more dangerous for old age groups? *	312 (79)	83 (21)
Are children at high risk to develop COVID-19 complications? *	217 (54.9)	178 (45.1)
People with chronic diseases (Hypertension, diabetes, heart disease and respiratory system) are at high risk to develop COVID-19 complications. *	359 (90.9)	36 (9.1)
People with severe obese are at high risk to develop COVID-19 complications. *	177 (44.8)	218 (55.2)
The disease is more dangerous in people with weakened immune systems *	346 (87.6)	49 (12.4)
Eating or contacting wild animals would result in the infection by the COVID-19 virus. *	200 (50.6)	195 (49.4)
Eating garlic, onions and pickles is an optimal method for preventing COVID-19 infection. **	203 (51.4)	192 (48.6)
Is wearing masks prevent COVID-19 infection? *	356 (90.1)	39 (9.9)
To prevent infection by COVID-19, an individual should avoid going to crowded places*	346 (87.6)	49 (12.4)
Is COVID-19 have specific treatment? **	157 (39.7)	238 (60.3)
Is mortality and morbidity rate from COVID-19 infections increase globally? *	357 (90.4)	38 (9.6)

**Note**:

* indicates true;

** indicates false answer (s)

### Student’s attitudes towards COVID-19

Slightly more than ¾ (76.5%) of the study participants agreed that the consequences of COVID-19 illness are serious. Two hundred eighteen one (71.1%) of the participants thought that COVID-19 preventative measures should be applied by everyone regardless of their status. Besides, 87.3% of the participants believed that COVID-19 suspected cases should be reported to health authorities to seek medical assistance. Moreover, 304 (77%) of participants agreed that COVID-19 is a preventable disease. In addition, 166 (42.2%) of the participants were agreed that preventative measures taken by the Ethiopian government at the beginning of the COVID-19 pandemic is not sufficient and timely. Overall, 86.3% (95% CI: 83–90) of study participants had a positive attitude towards COVID-19 infection (Table 3).

**Table 3:** Frequency of respondent’s attitude towards COVID-19, among secondary school students in Gondar town, Northwest Ethiopia, 2021.

Questions/statements	Agree N (%)	Disagree N (%)	Neutral N (%)
Do you think that COVID-19 prevention measures should only be applied by older adults and groups most at risk?	76(19.2)	281(71.1)	38(9.6)
Do you believe the consequences of illness are serious?	302(76.5)	48(1.2)	45(11.4)
Do you think it is crucial to report a suspected case to health authorities?	345(87.3)	31(7.8)	19(4.8)
Do you think that people suspected to have COVID-19 should be quarantined?	352(89.1)	31(7.8)	12(3.0)
Do you think wearing face mask in crowded place (school, spiritual holiday, cinema) is important?	346(87.6)	38(9.6)	11(2.8)
Do you think wash hands and face after coming outsides is good?	348(88.1)	33(8.4)	14(3.5)
Do you think COVID-19 is a preventable disease?	304(77.0)	56(14.2)	35(8.9)
Do you think COVID- 19 can be treated at home?	210(53.2)	121(30.6)	64(16.2)
Do you think health education can play an important role in COVID-19 prevention?	311(78.7)	58(14.7)	26(6.6)
Do you think that the preventative measures taken by the Ethiopian government in the beginning were sufficient?	142(35.9)	166(42.0)	87(22.0)
Do you think COVID-19 virus finally will be successfully controlled by the Ethiopian government?	103(26.1)	200(50.6)	92(23.3)
Do you think social distancing is important to prevent COVID-19?	330(83.5)	44(11.1)	21(5.3)
Do you believe the epidemic affects you academic performance?	299(75.7)	65(16.5)	31(7.8)
Do you believe there are some foods that can effectively cure or prevent COVID-19?	105(46.8)	109(27.6)	101(25.6)

### Student’s preventive practices towards COVID-19

**Fig 2:**
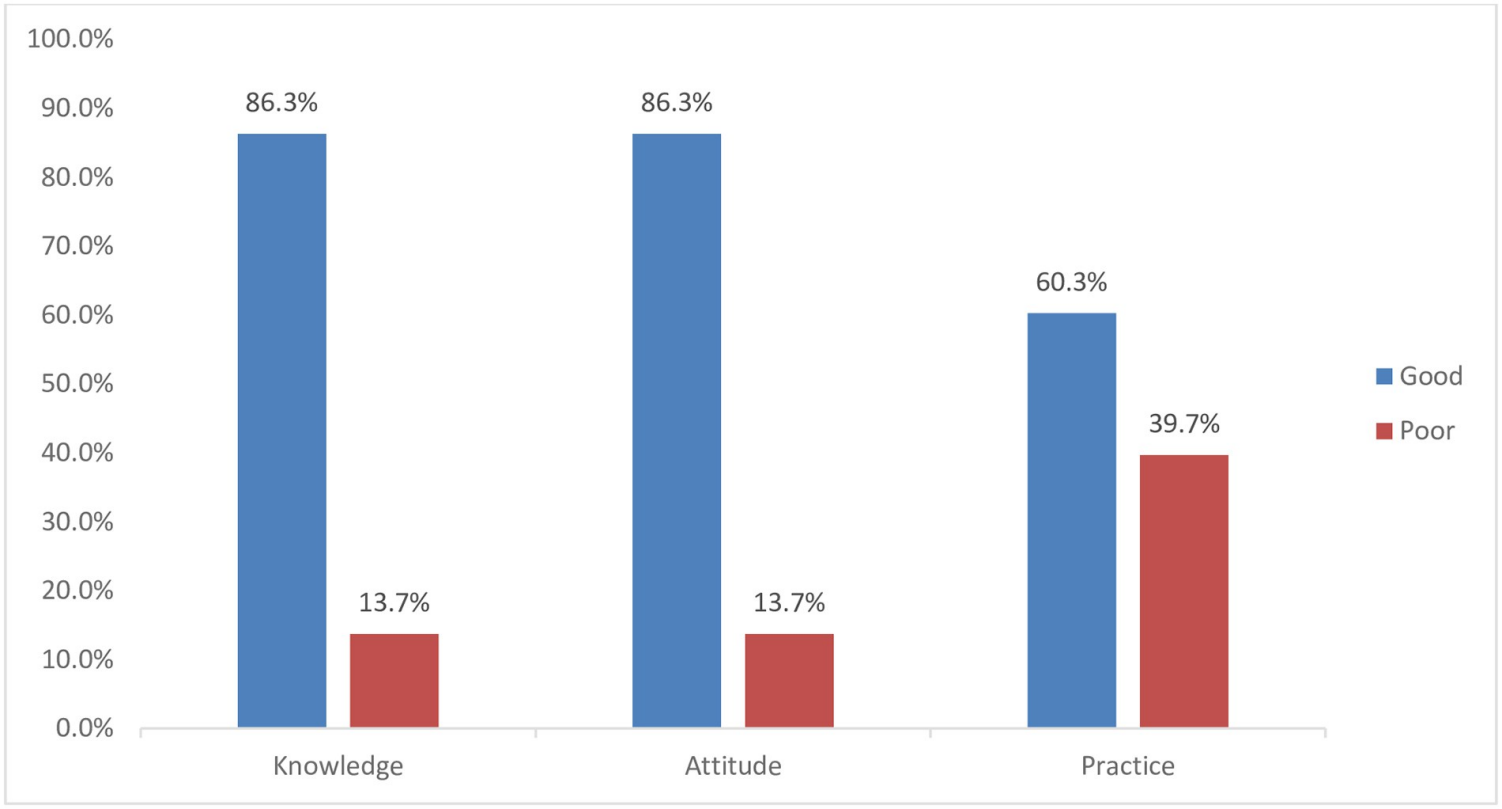
Overall KAP towards COVID-19 among secondary school students in central Gondar zone, Northwest Ethiopia, 2021.

There are 12 questions to evaluate the student’s preventive practices towards COVID-19. The majority, 302 (76.5%) of participants had regular practice of hand washing using water and soaps. Besides, 209 (52.9%) and 244 (56.7%) of the participants don’t keep social distancing and had not stopped unnecessary public transportation and travels in recent days, respectively. Two hundred fifty-six (64.8%) of the participant’s wore mask when leaving home, 143 (36.2%) did not avoid handshaking and kissing with someone, and 195 (49.4%) did not apply government preventive rules related to the COVID-19. Overall, 238 (60.3%) of the participants had good practice to prevent COVID-19 infections (Table 4, Fig 2).

**Table 4:** Preventive practice of secondary school students towards COVID-19 in Gondar town, Northwest Ethiopia, 2021.

Questions/statements	Yes N (%)	No N (%)
Do you wash hands regularly using water and soaps? *	302(76.5)	93(23.5)
Do you use a face mask when you left the house (crowds’ area and healthcare settings)? *	256(64.8)	139(35.2)
Do you maintain social distance between you and the individuals around you? *	186(47.1)	209(52.9)
Do you avoid hand shaking and kissing with any one? *	143(36.2)	252(63.8)
Do you use tissues or hanker chips during coughing/sneezing? *	243(61.5)	152(38.5)
Do you use alcoholic hand rub (sanitizer)? *	232(58.7)	163(41.3)
Do you avoid touching face, eyes and nose through contaminated hand? *	178(45.1)	217(54.9)
Do you eat healthy food focusing on outbreak? (vitamin and nutritional supplements) *	228(57.7)	167(42.3)
Do you maintain a healthy lifestyle focusing on outbreak? Including Sport activities *	215(54.4)	180(45.6)
Do you avoid Public Transportation and unnecessary travel? *	171(43.3)	224(56.7)
Do you taking action when symptoms of COVID-19 occur (quarantine, visit health facilities)? *	118(29.9)	277(70.1)
Do you obey all government rules related to the COVID-19? *	200(50.6)	195(49.4)

Note:

* indicates true answer (s)

### Factors associated with knowledge regarding to COVID-19

In this study, bivariate logistic regression analysis showed that variables like gender, residence, religion, source of information, occupation, and educational status of their father were associated with the level of knowledge about COVID-19 among secondary school students. Then, those variables with a p-value less than 0.2 were fitted to multivariate logistic regression analysis. Accordingly, after adjusting potential confounding variables, multivariate analysis affirmed that being urban residency (AOR: 5.6; 95% CI: 1.76–17.6), having a father with a diploma and above educational status (AOR: 3; 95% CI: 1.2–7.5) and students who use television or radio as a source of information (AOR: 3.7; 95%CI: 1.5–9.3) were significantly associated with good knowledge about COVID-19 among study participants (Table 5).

**Table 5:** Factors associated with knowledge about COVID-19 among secondary school students in Gondar town, Northwest Ethiopia, 2021.

Characteristics	Category	Knowledge		COR (95% CI)	AOR (95% CI)
		Good N (%)	Poor N (%)		
Age/years	14–16	66(83.5)	13(16.5)	0.6(0.19–1.69)	-
	17–19	236(86.5)	36(13.5)	0.7(0.26–1.91)	-
	20–23	45(90)	5(10)	1^a^	-
Gender	Male	121(82.3)	26(17.7)	1^a^	1^a^
	Female	220(88.7)	28(11.3)	0.6(0.33–1.06)	0.6(0.31–1.09)
Grade	9 and 10	147(86.5)	23(13.5)	1.0(0.52–1.83)	-
	11 and 12	194(86.2)	31(13.8)	1^a^	-
Resident	Urban	329(87.3)	48(12.7)	3.4(1.23–9.56)	5.6(1.76–17.6)*
	Rural	12(66.7)	6(33.3)	1^a^	1^a^
Religion	Orthodox	276(85.2)	48(14.8)	1.9(0.77–4.59)	0.5(0.18–1.18)
	Muslim and others	65(91.5)	6(8.5)	1^a^	1^a^
Father’s educational status	No education	95(89.6)	11(10.4)	1^a^	1^a^
	Primary/secondary	150(88.8)	11(11.2)	2(1.02–3.80)	2.2(1.02–4.80)*
	Diploma and above	96(80)	24(20)	2.2(1.00–4.65)	3.0(1.20–7.50)*
Father’s occupation	Government employed	100(82.6)	21(17.4)	0.4(0.15–1.03)	0.6(0.21–1.85)
	Not employed	169(86.2)	27(13.8)	0.5(0.21–1.32)	0.5(0.2–1.4)
	Others	72(92.3)	6(7.7)	1^a^	1^a^
Source of information about COVID-19	Television /radio	276(88.7)	35(11.3)	3.1(1.32–7.20)	3.7(1.50–9.30)*
	Social media/internet	42(80.8)	10(19.2)	1.6(0.58–4.62)	2.2(0.71–6.7)
	Others	23(71.9)	9(28.1)	1^a^	-
Family member >60 years old?	Yes	144(88.3)	19(11.7)	0.7(0.41–1.35)	-
	No	197(84.9)	35(15.1)	1^a^	-
Number of family members	≤5	157(87.7)	22(12.3)	1^a^	-
	>5	184(85.2)	32(14.8)	0.8(0.45–1.44)	-

**Note**:

* indicate statistically significant at P ≤ 0.05,

1^a^ indicates reference category

**Abbreviation**: COR; Crude Odd Ratio, AOR; Adjusted Odd Ratio, CI; Confidence Interval

## Discussion

The COVID-19 pandemic contributes a major global challenges and burdens, such as the closure of schools, political crises, economic crises, a burden on healthcare providers, and considerable public reaction [21]. This study enrolled a total of 422 participants to identify the current status of knowledge, attitude, and practice regarding COVID-19 among secondary school students in Gondar town.

In this study, 86.3% (95% CI: 83–90) of study participants had good knowledge about COVID-19. This high level of good knowledge is very encouraging since correct and adequate knowledge is the key to keeping oneself and others safe [22]. The finding of this study is lower than a study conducted in Egypt [23] and India [24] that reported 90% and 90.4% of students had good knowledge about COVID-19, respectively. However, it is higher than a study conducted in china indicating 74.1% of students acquired a certain understanding of COVID-19 [25]. The good knowledge of students towards COVID-19 in this study might be due to the late confirmed case report of COVID-19 in Ethiopia, which might provide adequate time to know about the disease. Besides, the WHO declaration of the disease as a pandemic might also have increased the students’ knowledge [26, 27]. Effective education by the WHO and the Ethiopian government and also active information seeking of students about COVID-19 from various channels of information and the official website of the Ministry of health of Ethiopia might contribute to good knowledge of students about COVID-19.

Moreover, 90.9% of study participants are aware that patients with the compromised immune systems and chronic diseases are at high risk of developing COVID-19 complications. The elderly people with co-morbidities were reported to be at the highest risk of contracting the disease and needed special care. These results support the curative approach, whereby the treatment would probably be established early in case of symptoms and the preventive approach needs further strengthening [28]. Almost all the study participants (94.9%) and (94.7%) reported that COVID-19 is a viral disease that affects the respiratory system and transmitted by airborne through respiratory droplets, respectively. The finding of this study is following a study conducted in Palestine [29] and Italy [30] in which 97.6% and 71% of students responded COVID-19 is a viral disease, respectively.

According to the present study, students with urban residency were 5.6 (AOR: 5.6; 95%CI: 1.76–17.6) times more knowledgeable than rural students. This result is similar to the finding of a study conducted in China [25]. This might be because students who were in urban areas had a higher chance of access to the main sources of information including television, radio, and social media [31]. The availability of adequate infrastructures to facilitate health education by health care professionals and also government officials also might contribute to the good knowledge of students.

This study also showed that students who use television or radio as a source of information were 3.7 times (AOR: 3.7; 95% CI: 1.5–9.3) more knowledgeable about COVID-19 than their counterparts. This might be because government media might provide reliable information to participants, and those who heard information from friends might hear rumors that may confuse them [32]. Social media (13.2%) is the second most preferred source of knowledge followed by family members (4.6%). This may be due to ease of access to readily updated information to most students via the internet and social media. This indicates the importance of the Internet in health promotion, especially during the occurrence of pandemics [33]. Online information becomes one of the principal and rapid ways to obtain information, compared with other resources [23]. The result of this study are consistent with those studies conducted in Egypt [30] and Ethiopia [26] that reported internet and social media as a main source of information. Against other media, newspaper, religious and community leaders, and heath extension workers appears to be a less favored option for gathering knowledge about the disease. Therefore, the health system of Ethiopia could improve the involvement of health extension workers and religious and community leaders to effectively disseminate information to the public.

The findings of this study suggest that students having a father with a diploma and above educational qualifications (AOR: 3; 95% CI: 1.2–7.5) had good knowledge about COVID-19 than their counterparts. Due to the previous closure of the school, the majority of the students were living with their families across the country. This might create the chance for strict guidance and delivery of adequate information about COVID-19 by the family members to the students. This is confirmed by the finding of the current study in that family members were their preferred source of information about the pandemic next to the television and social media.

About the attitude of students towards COVID-19, more than 3/4 (76.5%) of the study participants agreed that the consequences of COVID-19 illness are serious. A majority (71.1%) of the participants thought that COVID-19 preventative measures should be applied by everyone regardless of their status. This study is analog with previous studies in Egypt [34], Palestine [29], and Pakistan [35]. Besides, 87.3% of the participants believed that COVID-19 suspected cases should be reported to health authorities to seek medical assistance. Moreover, 304 (77%) of participants agreed that COVID-19 is a preventable disease. These findings were similar to a study conducted in Bangladesh [36].

A majority (75.7%) of the study participants believe that the COVID-19 pandemic affects their performance in their education. The finding of this study is higher than a study conducted in China [25] in which 55.0% of the students believed that the epidemic will affect their academic performance. Even though COVID19 is primarily affecting public health, spillover effects can already be observed in education, stemming largely from extended school closures. On the other hand, extended interrupted education that disengages students from the learning process has the potential cost of reversing gains in learning results [37].

In terms of preventive measures, 238 (60.3%) of the participants had favorable practices to prevent COVID-19 infections. The majority of the students were agreed in following good practices against COVID-19 such as regular hand washing with soap and water (76.5%) and (64.8%) of them wore masks when leaving home. This study is in corroborates with a population-based study in Iran [19], Beijing [25], and Italy [30]. The WHO has adopted frequent hand washing, wearing a face mask, and social distancing as effective measures to prevent the spread of COVID-19 infection [38, 39]. Pieces of evidence suggest that wearing a face mask has paramount importance to prevent infection [40]. At the start of the COVID-19 pandemic, there were inconsistencies regarding the advice for face masks. There was a scarcity of masks initially, however, appreciable efforts were subsequently made by the Ethiopian government to ensure the wide availability of masks to the public. The government also declared that wearing masks is mandatory in public places of Ethiopia. These factors might contribute to the good knowledge of students.

Meanwhile, 157 (39.7%) of the study participants had unfavorable practices towards COVID-19 infection. Two hundred nine (52.9%) and 244 (56.7%) of the participants do not keep social distancing and had not stopped in unnecessary public transportation and travels in recent days, respectively. Moreover, 36.2% did not avoid hand shaking kissing with someone practice, and 49.4% did not apply government preventive rules related to the COVID-19. This is supported by previous studies suggesting that adolescents are more likely to engage in risk-taking behaviors [41]. The finding of this study contradicts the study in Palestine [29]. Due to limited access to online health information and mass media, we worried that vulnerable populations with no formal education, geriatric and rural populations would be more likely to have poor knowledge, attitudes, and preventive practices. This indicates priority should be given to improving prevention practices rather than health education and delivering protective equipment to the public.

### Limitation of the study

Based on the used sample sizes the result could not be generalized to all the populations of Gondar and Ethiopia as well, even if the study can certainly help the state and the country to enhance the awareness regarding KAP in the general population. There is also a high chance of misrepresentation of information and recall bias by the participants due to the self-administered nature of the questionnaire. Moreover, the cross-sectional nature of the study did not allow to investigate the cause-effect relationship.

## Conclusion

Majority of the secondary school students in Gondar town have good KAP towards COVID-19. However, targeted interventions are still necessary, especially for students having poor knowledge and unfavorable practice towards COVID-19. Our study also found that urban residence, father with diploma and above educational status, and source of information about COVID-19 were significantly associated with the knowledge of the study participants. Besides, limited studies were found in this area to compare the finding of the study; further investigation may be necessary for better elaboration of the problem.

## Abbreviations

COVID 19: Coronavirus Disease 2019
CSA: Central Statistics Agency
KAP: Knowledge Attitude and Practice
PPE: Personal Protective Equipment
SARS: Severe acute respiratory syndrome
SARS-CoV-2: Severe Acute Respiratory Syndrome Coronavirus 2
WHO: World Health Organization
